# Children's physical activity levels during school recess: a quasi-experimental intervention study

**DOI:** 10.1186/1479-5868-4-19

**Published:** 2007-05-21

**Authors:** Nicola D Ridgers, Gareth Stratton, Stuart J Fairclough, Jos WR Twisk

**Affiliations:** 1 Research Institute for Sport and Exercise Sciences, Liverpool John Moores University, Henry Cotton Campus, 15-21 Webster Street, Liverpool L3 2ET, UK; 2 Centre for Physical and Outdoor Education, Liverpool John Moores University, I.M. Marsh Campus, Barkhill Road, Liverpool, L17 6BD, UK; 3 The REACH Group, Liverpool John Moores University, Liverpool, UK; 4 Department of Clinical Epidemiology and Biostatistics, VU University Medical Centre, Amsterdam, The Netherlands; 5 Department of Methodology and Applied Biostatistics, Institute of Health Services, Vrije University, Amsterdam, The Netherlands

## Abstract

**Background:**

Recess provides a daily opportunity for children to engage in moderate-to-vigorous (MVPA) and vigorous physical activity (VPA). Limited research has investigated the effects of recess-based interventions on physical activity using large sample sizes whilst investigating variables that may influence the intervention effect. The aim of the study was to investigate the short-term effects of a playground markings and physical structures intervention on recess physical activity. A secondary aim was to investigate the effects of covariates on the intervention.

**Methods:**

150 boys and 147 girls were randomly selected from 26 elementary schools to wear uni-axial accelerometers that quantified physical activity every 5 seconds during recess. Fifteen schools located in deprived areas in one large urban city in England received funding through a national initiative to redesign the playground environment. Eleven schools served as matched socioeconomic controls. Data were collected at baseline and 6-weeks following playground intervention. Recess MVPA and VPA levels adjusted for pupil- and school-level covariates (baseline physical activity, age, gender, recess length, body mass index) were analysed using multilevel analyses.

**Results:**

Positive but non-significant intervention effects were found for MVPA and VPA when confounding variables were added to the model. Gender was a significant predictor of recess physical activity, with boys engaging in more MVPA and VPA than girls. Significant interactions for MVPA revealed that the intervention effect was stronger for younger elementary aged school children compared to older children, and the intervention effect increased as daily recess duration increased.

**Conclusion:**

The playground redesign intervention resulted in small but non-significant increases in children's recess physical activity when school and pupil level variables were added to the analyses. Changing the playground environment produced a stronger intervention effect for younger children, and longer daily recess duration enabled children to engage in more MVPA following the intervention. This study concludes that the process of increasing recess physical activity is complex when school and pupil-level covariates are considered, though they should be taken into account when investigating the effects of playground intervention studies on children's physical activity during recess.

## Background

Physical activity is an integral component of a healthy lifestyle. Engaging in regular physical activity during childhood is hypothesized to reduce the health risks associated with inactivity and benefit health both during childhood and adulthood [1,2]. In recent years considerable attention has focused on determining habitual physical activity levels in children [3,4]. Activity guidelines have recommended that children accumulate sixty minutes of at least moderate intensity physical activity a day [5,6]. Whilst there is some empirical data that indicate many children are achieving this target [3,4], concern remains that a large proportion of children are insufficiently active in order to gain subsequent health benefits [7,8]. Since physical activity levels have been found to decrease across childhood and adolescence into adulthood [9], and that physical inactivity tracks across time [10], the promotion of health enhancing physical activity to children has become a public health priority.

The school has been identified as a key setting for the promotion of physical activity to young people [2,11]. School attendance is a generic part of childhood; therefore schools provide a conceivable and logical setting for physical activity promotion. Physical education (PE) has been the traditional backdrop for promoting physical activity in school time, though there is concern that PE is unlikely to provide sufficient activity for notable health benefits to be accumulated [7]. A recent review indicated that if PE was offered on a daily basis, which in the United Kingdom (UK) it is generally not, PE could contribute around one fifth towards daily activity guidelines [12]. An alternative but complementary setting to PE for providing daily physical activity opportunities at school is recess [13]. In the UK, children experience up to 600 recess periods a year (based on 3 times a day, 5 days a week, 39 weeks a year; [14]), therefore offering a significant amount of time where children can engage in moderate-to-vigorous physical activity (MVPA) and vigorous physical activity (VPA; [13]). In light of this, the efficacy of recess-based interventions in order to increase physical activity levels has been advocated [14-16].

Little empirical research has investigated the effects of recess-based interventions on children's recess physical activity levels [13]. Interventions conducted within this context include playground markings [14,15,17], activity and fitness breaks [18-20] games [20] and games equipment [16], which have all documented significant increases in recess physical activity [14-16,18-21] and energy expenditure [17], albeit using generally small sample sizes over short-term follow-ups. A common element of these studies has been reporting the effects of recess interventions on boys and girls' physical activity to determine whether the effects were different for either gender [15,16,19,20]. However, a number of moderating variables may influence the outcome of a recess physical activity intervention [22]. For example, Verstraete et al [16] found that boys and girls' physical activity levels decreased during morning recess 3 months after the intervention was implemented in a short morning recess, though activity increased during the lunch recess. This suggests that intervention may be effective when the recess is of sufficient duration. Few studies however have explored variables that may moderate any intervention effects. Therefore, the purpose of this study was twofold: firstly, to investigate the impact of a playground redesign intervention on children's MVPA and VPA using accelerometry, and second, to determine the potential influence of pupil-level and school-level moderating variables on the intervention effect.

## Methods

### Participants and settings

One hundred and fifty boys and 147 girls randomly selected from 26 elementary schools from one large urban city in the North West of England returned signed parental informed consent to participate in the study. Eleven children per elementary school (stratified by gender) were recruited equally from Grades K-1 (age 5–7 years) and Grades 2–4 (age 7–10 years). All children who were randomly selected from the schools participated in the project. Fifteen schools (76 boys, 73 girls) took part the intervention, and 11 schools (74 boys, 74 girls) served as socioeconomic matched controls.

All schools were located in one Local Authority which was involved in a national £10 million Sporting Playgrounds' initiative. Through the initiative, funding was allocated to the Local Authority to improve the playground environment of schools situated in areas of high social and economic deprivation. To be involved in the project, schools had to meet two criteria that aimed to identify schools in deprived areas that lacked adequate playground facilities. First, schools had to be included within a School Sport Partnership, which aim to develop PE and sport opportunities for young people across groups of UK schools. This enabled the partnerships to identify schools that had no markings or physical structures, and to provide support to the schools for the use of the facilities beyond recess. Second, schools had to be located within a Sport Action Zone (SAZ). SAZs are located in areas of high deprivation across the UK and aim to address sporting deprivation in these areas, and integrate sport in to local health, education and social inclusion agendas. SAZs were created in areas of high deprivation, where children are at risk of poor physical health and low education attainment, as their families lack the resources that enable them to access activities that can benefit health and education, for example [23]. Identified schools were then ranked on socioeconomic status and indices of deprivation. Thirty schools were identified as meeting these criteria, and 26 schools agreed to participate in the study (86%). Each intervention school received £20,000 to redesign the playground environment [24]. No schools participating in the project had playground markings prior to the study commencing.

### Measures

#### Physical activity

Children's physical activity levels during recess were quantified using accelerometry. The ActiGraph (Model 7164, MTI Health Services, Florida, USA) is a uni-axial accelerometer that measures vertical acceleration of human motion. The detected accelerations are filtered, converted to a numerical value and subsequently summed over a specified time interval or epoch prior to the commencement of data collection [25]. The recorded counts for each epoch represent the intensity of the activity undertaken during that time period. At the end of each epoch, the summed value is stored in the memory and the ActiGraph is automatically reset to zero [25]. The epoch time length for the current study was set at 5 s [26].

Activity count thresholds were used to determine the amount of time the children engaged in physical activity at moderate, high and very high intensities [26]. These were represented by 163–479, 480–789, and ≥ 790 counts per 5 s epoch respectively [26]. The amount of time children spent in MVPA during recess was determined by summing the amount of time spent in each of the physical activity intensities. VPA was determined by summing high and very high intensity physical activity. The relative (percentage) time spent in MVPA and VPA during recess were calculated and used in the subsequent analyses. Of the initial 297 children who were monitored at baseline, post-test measures were recorded for 242 children (106 intervention, 136 control). Twenty children's data (9 boys, 11 girls) were lost due to monitor malfunction. Thirteen children were absent from school at the 6-week follow-up measurement (7 boys, 6 girls), and 22 children (11 boys, 11 girls) had left the schools being monitored.

#### Anthropometry

Measurements of stature (to the nearest 0.1 cm) and body mass (to the nearest 0.1 kg) were recorded using the Seca scales (Seca Ltd, Birmingham, UK) and the Leicester Height Measure (Seca Ltd, Birmingham, UK). Children were measured without footwear whilst wearing minimal school uniform (trousers/skirt, shirt) prior to the fitting of the physical activity monitoring equipment at the start of the school day. In order to determine whether the children were normal weight, overweight or obese for their age, the measurements of body mass and stature were used to determine each child's BMI. BMI was calculated using (weight (kg)/height^2 ^(m)). Children were classified as normal weight, overweight or obese using age specific international cut off points [27].

#### Demographic characteristics

Data on children's sex were collected using class lists provided by the schools once they had agreed to participate in the study. This enabled the stratification of the sample. Parents reported the date of birth of participating children in the informed consent form.

#### Recess duration

Recess time was defined as the time the school bell rang to start recess to the time it rang to conclude recess. Data on recess duration was collected by the principal researcher who recorded the time that the school bell rang to start and conclude each recess period in all participating schools.

### Procedure

Schools were visited twice during this study, once prior to the playground redesign, and once 6 weeks following the playground redesign (post-test). Schools suggested data collection days to ensure that children would not be out of school on external trips, or undertaking formal examinations or inspections. The visits formed part of a longitudinal study, which is investigating the long-term impact of a playground environment intervention on children's recess physical activity levels and play behaviors. As a consequence, children in the last year of elementary school were excluded from the project, as they would not be available for the longitudinal component of the study. During each testing day, the randomly selected children underwent a familiarization period with the monitoring equipment at the start of the school day. All children then followed their normal daily school routine, with physical activity being monitored during morning, lunch and afternoon recess periods. Monitors were removed at the conclusion of the last recess of the day. A member of school staff supervised the fitting and removal of the monitors alongside the principal researcher. The research protocol and design received ethical approval from the Liverpool John Moores University Ethics Committee.

Each intervention school received new multicolor playground markings and physical structures where the playground area was divided into three colored zones. The zones were a sports area (Red Zone), a fitness and skills area (Blue Zone) and a 'chill out' area (Yellow Zone). The markings contained in each zone were relevant to the physical activity and social behaviors desired for that area. For example, the red sports zone contains markings for soccer, tennis and basketball (for more information, see [24]). Schools were encouraged to explain the aims of the zones to the children through class time, and reinforce these aims during the recess periods. This was conducted prior to the follow-up visit by the research team. The control schools received no playground redesign through the study. Small pieces of sports equipment such as skipping ropes and soccer balls were available for use in all school playgrounds throughout the study. Schoolteachers supervised morning and afternoon recess, whilst specially employed lunchtime assistants supervised lunch recess in both the intervention and control schools. No supervisors received training in the promotion of playground physical activity.

Eleven children per school wore an accelerometer for one school day at each measurement period. Baseline measures were collected between July 2003 and March 2004. Intervention phase data were collected 6-weeks following the painting of each of the playgrounds between March 2004 and July 2004, with the average time lapse between measures being 6 months. Control school data were collected during these two measurement periods, with the timeframe between measures matched for the intervention and control schools. Seasonality and day of data collection were not controlled for, as a previous study conducted geographically close to the current schools found no significant differences in children's recess physical activity levels between days and seasons [28].

Accelerometers were fitted to the children's right hip using a tightly fitted elastic belt following a familiarization period where children became accustomed to the monitors. During this time children were asked to follow their normal daily school routine. The monitors were worn during morning, lunch, and where applicable, afternoon recess. During this time children were instructed to seek the researchers for refitting if the monitors became detached. Children were then asked to follow their normal daily routine. Monitors were removed at the end of the school day and the data immediately downloaded using a reader interface unit connected to a computer and analyzed using the ActiSoft Analysis Software Version 3.2 (MTI Health Services, Florida, USA). These procedures were repeated at each phase of the study.

### Data analysis

Descriptive statistics (mean ± SD) were calculated to describe the anthropometric characteristics of the children. Exploratory independent t-tests were conducted to examine gender and intervention group differences in baseline variables. In addition, due to the attrition rate, independent t-tests were used to explore potential differences in children's recess physical activity levels at baseline between children retained in the study and those who dropped out. Descriptive data were analyzed using the Statistical Package for the Social Sciences version 12 (SPSS Inc., Chicago, IL, USA).

The main analysis used in this study to estimate the effect of the intervention on children's recess physical activity was multilevel modeling, which is considered to be the most appropriate data analysis technique for nested data [29]. In this study, a two-level data structure was used where children were defined as the first level unit and schools as the second level unit [30]. School was included as a second level unit to control for the effect that a particular context, such as school, can have on a child's behavior [30]. The data were analyzed using MLwiN 1.10 software (Institute of Education, University of London, UK). An association model approach was used to determine the effects of the intervention after being corrected for confounding variables [30]. MVPA and VPA 6-weeks following the intervention were the outcome variables, with baseline values for recess physical activity, body mass index (BMI), age and daily recess time (continuous variables) and gender (dichotomous variable) being used as covariates. Two analyses were conducted for the outcome variables on the 6-week follow-up measurement. The first analysis determined the difference between the intervention and the control group on children's recess physical activity levels on the follow-up measure ('crude' analysis), whilst the second determined this effect when the covariates were added to the model ('adjusted' analysis; [30]). In addition, potential effect modification was assessed for all covariates in order to investigate whether the intervention effect is different for different subgroups. This was assessed using interaction terms, which consisted of the intervention effect and the covariate. Interaction terms were added separately to the analyses to determine their effects on the effect of the intervention [30]. Separate analyses were conducted for MVPA and VPA. Regression coefficients in the model were assessed for significance using the Wald statistic [30]. Statistical significance was set at *P *< 0.05, with the exception being interaction terms where it was *P *< 0.10 [30].

## Results

### Exploratory analyses

Independent t-tests revealed no significant differences between complete and incomplete measures in baseline recess physical activity for boys and girls (*P *> 0.05).

### Main analyses

The descriptive (mean ± SD) anthropometric characteristics and baseline physical activity levels of the children are shown in Table 1. Independent t-tests revealed that the experimental boys had greater stature, body mass and BMI, but engaged in lower levels of VPA during recess than the control boys at baseline (*p *< 0.05). No significant differences were found on the anthropometric data for the girls, though experimental girls engaged in significantly less MVPA and VPA during recess than control girls (*p *< 0.01). The mean daily recess time available for children to engage in physical activity was 81.1 (± 17.3) minutes (range = 31–140 minutes).

**Table 1:** Descriptive baseline anthropometric and physical activity data (mean ± SD)

	**Boy**		**Girl**	
	
**School**	**Exp**	**Con**	**Exp**	**Con**
**Age (Years)**	8.3 ± 1.8	7.8 ± 1.5	8.3 ± 1.9	7.9 ± 1.4
**Stature (m)**	1.33 ± 0.09*	1.30 ± 0.09*	1.31 ± 0.09	1.29 ± 0.11
**Body Mass (kg)**	32.6 ± 8.1*	28.9 ± 7.9*	30.5 ± 8.2	29.7 ± 9.1
**Body Mass Index (kgm**^-2^**)**	18.2 ± 2.9*	16.8 ± 2.7*	17.6 ± 3	17.6 ± 3
**MVPA (% recess)**	30.8 ± 11.7	33.1 ± 13.7	21.9 ± 9.9^	27 ± 10.4^
**VPA (% recess)**	4.5 ± 4.1^#^	7 ± 5.7^#^	2.9 ± 3.6^	6.5 ± 4.9^

* Significant t test intergroup result: Experimental boys < Control boys; *p *< 0.05

^# ^Significant t test intergroup result: Experimental boys > Control boys; *p *< 0.05

^ Significant t test intergroup result: Experimental girls > Control girls; *p *< 0.01

Table 2 shows the effect of the intervention on MVPA at the 6-week follow-up measure. A statistically significant effect was found for the intervention, with the intervention group engaging in 5.95% (CI: 0.14 to 11.77) more MVPA during recess than the control group respectively (crude analysis). When the correction for potential confounders was performed (adjusted analysis), the regression coefficient for the intervention term was reduced and rendered non-significant for both MVPA. The analyses revealed however that sex and BMI were significant negative exploratory variables of recess MVPA following the intervention. The results indicate that boys engage in 7.2% more physical activity than girls during recess. In addition, the results indicated that as BMI increased, recess physical activity decreased, though this change was relatively small.

**Table 2:** Results of multilevel model analysis on the intervention on children's recess MVPA (% of total recess)

	**Model 1**			**Model 2**		
	
	**β (SE)**	**95%CI**	***p***	**β (SE)**	**95%CI**	***p***
**Constant**	14.20 (3.04)	8.24 to 20.16	<0.001**	23.88 (9.22)	5.81 to 41.94	0.009**
Baseline MVPA	0.49 (0.07)	0.36 to 0.62	<0.001**	0.37 (0.07)	0.24 to 0.49	<0.001**
Intervention	5.95 (2.97)	0.14 to 11.77	0.045*	4.5 (2.83)	-1.05 to 10.1	0.112
Sex	NE			-7.15 (1.42)	-9.94 to -4.36	<0.001**
Age	NE			-0.13 (0.62)	-1.34 to 1.09	0.841
BMI	NE			-0.55 (0.24)	-1.03 to -0.07	0.024*
Recess duration	NE			0.10 (0.07)	-0.04 to 0.24	0.147
						
**Random**						
School Level	40.15 (15.44)			35.45 (13.8)		
Child Level	125.17 (12)			99.10 (9.93)		
						
**Deviance**	1889.766			1698.943		

Abbreviations: MVPA = Moderate-to-Vigorous Physical Activity; CI = Confidence Interval; β = Regression coefficient; SE = Standard Error; NE = Not entered.

Model 1 is the crude analysis, where the intervention effect is controlled for baseline physical activity. For the intervention variable, the control group is the reference category). Model 2 is the adjusted analysis, where the effect of the intervention is corrected for sex (boy is the reference category), BMI, age and recess time. * *P *< 0.05; **P < 0.01.

Table 3 shows the effect of the intervention on both MVPA and VPA at the 6-week follow-up measure. A statistically significant effect was found for the intervention, with the intervention group engaging 1.7% (CI: 0.01 to 3.39) VPA during recess than the control group respectively (crude analysis). When the correction for potential confounders was performed (adjusted analysis), the regression coefficient for the intervention term was reduced and rendered non-significant for VPA. The analyses also highlighted that sex was a significant negative exploratory variable of recess VPA. Boys engaged in 3.1% more VPA than girls during recess.

**Table 3:** Results of multilevel model analysis on the intervention on children's recess VPA (% of total recess)

**Parameter**	**Model 1**			**Model 2**		
	
	**β (SE)**	**95%CI**	***p***	**β (SE)**	**95%CI**	***p***
**Constant**	4.63 (0.77)	3.12 to 6.14	<0.001**	9.69 (3.18)	3.46 to 15.92	0.002**
Baseline VPA	0.41 (0.07)	0.27 to 0.55	<0.001**	0.35 (0.07)	0.21 to 0.49	<0.001**
Intervention	1.70 (0.86)	0.01 to 3.39	0.049*	1.30 (0.79)	-0.24 to 2.84	0.098
Sex	NE			-3.05 (0.66)	-4.34 to -1.76	<0.001**
Age	NE			-0.09 (0.27)	-0.62 to 0.44	0.74
BMI	NE			-0.21 (0.12)	-0.45 to 0.03	0.08
Recess duration	NE			0.01 (0.02)	-0.03 to 0.05	0.617
						
**Random**						
School Level	1.19 (1.21)			0.74 (0.95)		
Child Level	28.48 (2.72)			23.4 (2.32)		
						
**Deviance**	1511.756			1347.832		

Abbreviations: VPA = Vigorous Physical Activity; CI = Confidence Interval; β = Regression coefficient; SE = Standard Error; NE = Not entered.

Model 1 is the crude analysis, where the intervention effect is controlled for baseline physical activity. For the intervention variable, the control group is the reference category). Model 2 is the adjusted analysis, where the effect of the intervention is corrected for sex (boy is the reference category), BMI, age and recess time. * *P *< 0.05; **P < 0.01.

An inverse interaction was found between the intervention and age for both MVPA and VPA (p = 0.01 and p = 0.09, respectively), indicating that the intervention effect is stronger for the younger children (Table 4). The effect was stronger for recess MVPA and VPA. In addition, a positive interaction was found between the intervention and daily recess time for MVPA (p = 0.07), indicating that the more daily recess time there is available, the stronger the intervention effect on recess MVPA. All other interactions (with baseline physical activity, gender and BMI) showed p-values > 0.10.

**Table 4:** Intervention interaction terms with covariates investigating potential effect modification

**Interaction term**	**β (SE)**	***p***
**MVPA**		
Intervention × MVPA baseline	-0.15 (0.14)	0.28
Intervention × sex	3.03 (2.75)	0.27
Intervention × age	-3.03 (1.19)	0.01**
Intervention × BMI	-0.13 (0.523)	0.81
Intervention × recess duration	0.25 (0.14)	0.07*
**VPA**		
Intervention × VPA baseline	-0.17 (0.16)	0.28
Intervention × sex	0.23 (1.31)	0.86
Intervention × age	-0.82 (0.48)	0.09*
Intervention × BMI	-0.02 (0.24)	0.93
Intervention × recess duration	0.02 (0.05)	0.74

* *p *< 0.1; ** *p *< 0.05

## Discussion

The purpose of the study was to evaluate the short-term effects of a playground redesign intervention on children's recess physical activity levels. A secondary aim of the study was to investigate the influence of pupil-level and school-level covariates on this effect, and how these variables interacted with the intervention effect.

The results indicate that the playground redesign intervention was effective in increasing children's MVPA and VPA in the short-term, when the intervention effect was evaluated separately, compared to control school children. The increases observed in the present study are smaller than studies that have investigated the short-term effects of playground markings on children's recess MVPA and VPA [14,15]. When potential pupil-level and school-level confounding variables were analyzed, small decreases were observed in the intervention regression coefficient, with the intervention effect for both MVPA and VPA becoming non-significant. These results highlight the influence of the assessed covariates on children's recess physical activity levels. Furthermore, it suggests that both individual and group-level variables affect children's physical activity during recess [31] and moderate the effects of recess interventions on physical activity [22]. Pellegrini and Smith [31] reviewed the effects of individual and group-level variables on children's behavior during recess, concluding that factors such as age, gender, and recess duration interact with each other and relate to playground activity and behavior. The findings from this study support this notion, and indicate that whilst the environmental intervention raised physical activity levels, the process of increasing activity during recess is complex when additional variables are considered. Future recess interventions should investigate the effects of moderating variables on intervention effects to determine the extent to which physical activity levels increase. In addition, recess interventions should consider a combination of initiatives targeting both individual factors and group factors to develop the utility of recess as a health promotion context.

Previous recess studies have evaluated the short-term effects of playground markings [14,15], fitness and activity breaks [18-20], equipment provision [16] and games on children's physical activity [21]. Some studies have compared the intervention effects between boys and girls' activity [14,18], or between children at different stages of schooling [15]. However, to the best of our knowledge, no empirical recess intervention studies have considered the effects that moderating variables including age, BMI and recess length have on physical activity levels.

A significant explanatory variable of recess physical activity in the adjusted models was sex, with boys engaging in significantly more MVPA and VPA during recess than girls. A recent review documented that cross-sectional studies have consistently reported that boys engage in more recess physical activity than girls [13]. Furthermore, recess intervention studies have reported that whilst boys and girls experience similar increases in their physical activity levels [15] and energy expenditure [17] boy's activity remains generally higher than the girls. Interestingly, the intervention effect by sex interaction term was non-significant when added to the multilevel model analysis, suggesting that there were no differences in the effect of the intervention on boys and girls' recess physical activity in the short-term. Whilst the underlying reasons for this finding are not known, it could be attributed to the markings within the zones not being designated as boys or girls' games [17]. The present study indicates that while boys are more active overall during playtime, boys and girls experienced comparable increases in their recess MVPA and VPA following the playground markings and physical structures intervention.

Significant interaction terms were found in the present study for age and recess length with the intervention effect. With reference to age, the intervention effect was stronger on younger children's MVPA and VPA compared to older children. This finding may be related to the social context of the school playground. Behavioral studies, both cross-sectional and longitudinal, have suggested that as children grow older, the size of their social group increases [32,33]. In addition, boy's networks are significantly larger than girls, and they are more likely to play with same-aged peers [33]. With older boys often dominating the available play space for active games such as soccer [34], younger children and older girls are often found on the margins of the playground [35]. The environmental intervention in this present study provided the intervention schools with both playground markings and physical structures, which increased the choice of activities during recess, potentially benefiting children in smaller social groups [33], and restricted competitive ball games (such as soccer) to one area. Pellegrini and Smith [31] noted that children were more active in spacious environments compared to restricted environments. The study found that younger children had more space and physical activity opportunities when soccer became less dominant. The use and availability of space on the playground may be an important factor in increasing children's recess physical activity levels. In light of this, further studies employing systematic observation of play behavior and use of the playground space are required to verify the findings from the present study.

A significant interaction for MVPA between the intervention effect and the recess length was found, with longer recess length increasing children's MVPA engagement. Previous studies have reported conflicting findings concerning physical activity engagement with regards to recess length. McKenzie et al [36] reported that as time elapsed at elementary schools, children became significantly less active. In comparison, Zask et al's [37] study highlighted that the length of recess contributed to recess physical activity engagement, with activity levels as a proportion of recess being higher during the longer lunch recess compared to morning recess. It was suggested that higher activity during lunch recess might reflect that the children had more time available to become engaged in games and other activities [37]. The present study lends support to this idea, as higher physical activity levels were associated with longer recess duration, suggesting that the intervention was more effective when recess was longer. Longer periods of recess time may enable children to become habituated to the activity opportunities on offer in the redesigned playgrounds [31]. A pressing concern in the recess literature has been the erosion of recess time available, with reductions being reported both in the UK and in the United States [38,39]. This study suggests that for playground interventions to be effective, longer recess periods are needed to have positive effects of children's recess physical activity.

Whilst the study examined the effects of a number of potential confounding variables, the study is limited, as it did not control the amount of equipment that was available to children in each school. Secondly, the method of supervision provided by the adult staff, for example actively prompting activity [36] or distant supervision, was not monitored. These factors may have effects on the physical activity levels of children during a recess intervention. It is recommended that future studies should combine physical activity measurements, for example accelerometry with direct observation, as the social context of the playground is an important influencing factor on children's recess physical activity and play behavior [31]. It is important to determine the effects of recess interventions on physical activity levels, but this study suggests that there is a need for observational data to explain what other factors may have influenced the findings.

This study focused on the effects of a school-based intervention on children's recess physical activity levels. However, the effect of the intervention on overall physical activity was not assessed. Previous studies that have investigated the physical activity levels of children during school time have reported mixed findings concerning physical activity levels outside of school time. Dale et al. [40] indicated that when minimal physical activity opportunities were provided during school time, children did not compensate by increasing their activity out of school, while active children during the school day were more active out of school. In contrast, Mallam et al. [41] reported that the children's total daily physical activity engagement did not depend on timetabled physical education as children compensated out of school hours. It is recommended that future recess studies should report the effects of school-based interventions on physical activity levels during recess alongside the effects on daily physical activity levels to determine whether the intervention promotes higher overall physical activity or has no effect on overall daily physical activity.

## Conclusion

The present study contributed to the dearth of empirical literature investigating the short-term effects of a school based intervention on children's recess physical activity levels and by looking at variables that may moderate the intervention effect. The study suggested that the effect of the intervention was not significant when potential confounders were added to the analysis. Interestingly, significant interaction terms indicated that the effects of the intervention were greater for younger children, and children with longer recess. It is recommended that future recess intervention studies combine direct observation with activity monitors to examine the social influences on children's physical activity levels. Moreover, there is a need to analyze recess intervention data using MLM analyses, which are appropriate for nested data. There is a need to evaluate the longer-term effects of environmental interventions on physical activity levels during recess.

## Competing interests

The author(s) declare that they have no competing interests.

## Authors' contributions

GS conceived the study and secured funding. NDR, GS and SJF contributed to the planning and design of the study. NDR collected the data. NDR and JWRT conducted data manipulation and analyses. NDR wrote the manuscript and GS, SJF and JWRT supplied comments. All authors read and approved the final manuscript.
